# Magnetic Microspheres as Microrobot Bodies: Optimized Chitosan Modification and Gel Dispersion for Controlled Release of Doxorubicin

**DOI:** 10.3390/mi17060696

**Published:** 2026-06-06

**Authors:** Shiqi Ma, Lizhong Xu

**Affiliations:** School of Mechanical Engineering, Yanshan University, Qinhuangdao 066000, China; mashiqi@stumail.ysu.edu.cn

**Keywords:** magnetic microspheres, surface modification, chitosan, optimized design, drug loading and release

## Abstract

Although the loading and targeted release of drugs are core biomedical applications of micro-nano robots, they are restricted by the complexity of robot fabrication and drug loading/release regulation. This work adopts magnetic microspheres (MMs) as microrobot bodies, owing to their low driving resistance and ease of preparation, in order to explore the loading and release of anticancer drugs via physical adsorption and chitosan surface functionalization. Two modification routes, chitosan solution (CS) and chitosan colloid (CC), were compared in terms of their efficacy in fabricating magnetic chitosan microspheres (MCMs). The dispersion procedure of chitosan gel (CG)-encapsulated MMs was optimized to obtain microspheres with uniform size and good encapsulation. Doxorubicin (DOX) was used as a model drug, and the optimized microstructure exhibited high loading efficiency and excellent controlled release. This study offers a low-cost strategy to advance micro-nano robots toward targeted drug delivery applications.

## 1. Introduction

Micro-nano robots have emerged as a highly promising frontier in biomedicine, mainly due to their unique size-dependent characteristics [1,2]. In physiological fluid environments, which are characterized by a low Reynolds number regime, generating net displacement requires the implementation of specialized locomotion mechanisms. Consequently, extensive research has been devoted to developing diverse actuation strategies, including physical field-driven [3], bubble-driven [4], chemical field-driven [5], and biologically inspired [6] approaches. Micro-nano robotic systems display a wide range of morphological architectures, such as spherical [7], disk-shaped [8], tubular [9], rod-shaped [10], and helical [11] geometries. More recently, advanced designs like beaker-like [12], cilia-inspired [13,14], film-based [15], and flagellum-mimicking [16] structures have further enriched the morphological diversity of these systems. From a hydrodynamic perspective, the spherical topology adheres to the principle of minimum drag, making it an optimal choice, and is often favored due to its structural simplicity [17]. However, the synthesis and operation of microspherical robots pose significant challenges, including complex fabrication processes [18], limited motion control [19], and a reliance on environmental resources for propulsion [20]. In this context, magnetic actuation has become a superior method, offering distinct advantages such as deep tissue penetrability, sub-millimeter precision, high maneuverability, and rapid dynamic response [21]. Additionally, magnetically responsive micro-nano robots can self-assemble into complex structures, such as chain-like [22], polymeric, and other non-standard configurations [23], enabling coordinated collective motion [24] without consuming ambient resources. These unique features make magnetic micro-nano robotic systems particularly appealing for in-depth research in the field of biomedical engineering [25].

Currently, micro-nano robots employ diverse drug delivery methodologies, such as the direct adsorption [26], encapsulation [27], chemical bonding [28], physical embedding [29], self-assembly [30], biomolecular conjugation [31], and magnetic drug delivery [32] techniques. The associated drug release mechanisms predominantly involve stimulus-responsive [33], diffusion-controlled [34], biodegradation-mediated [35], and electrochemical [36] processes. However, the existing drug loading procedures for these robots are often intricate, frequently requiring costly equipment. Moreover, preparation processes typically entail handling substantial amounts of toxic drugs, thereby posing significant safety hazards [37]. Additionally, the multi-step reactions involved in drug release mechanisms [38] render them challenging to characterize and control, contributing to a notable disparity between current technologies and practical clinical applications. Chitosan, a naturally derived cationic polysaccharide polymer, exhibits non-toxicity, biocompatibility, and biodegradability [39]. The amino and hydroxyl groups on its surface allow it to serve as an optimal carrier for drug molecules, leading to its extensive utilization in the biomedical field [40].

This study systematically investigated the doxorubicin (DOX) loading and release characteristics of magnetic microspheres (MMs) via physical adsorption and surface modification. To optimize MM surface functionalization, chitosan solution (CS) and chitosan colloid (CC) were synthesized and experimentally characterized. Two methods were used to prepare magnetic chitosan microspheres (MCMs) through CC-based surface modification: (1) adding sodium hydroxide solution to MM-dispersed CC; (2) introducing MM-dispersed CC suspensions into sodium hydroxide solution. To enhance the integrity and uniformity of the chitosan coating, key optimizations were applied to two processes: the dispersion of chitosan gel (CG)-coated MMs and the post-drying dispersion of chitosan particle-coated MMs. Finally, comprehensive DOX loading and release experiments were performed on unmodified MMs and surface-modified MCMs to systematically evaluate and compare the drug encapsulation efficiency and release kinetics of the two microcarriers.

## 2. Materials and Methods

The following experimental materials were used: Debai magnetic powder (Shandong Powder Metallurgy Family Powder Metallurgy Co., Ltd., Jinan, China), chitosan (degree of deacetylation: 80%, molecular weight (MV): 500 kDa; Shandong Keyuan Biochemical Co., Ltd., Jinan, China), doxorubicin hydrochloride (Hefei BASF Biotechnology Co., Ltd., Hefei, China), 0.01 mol/L PBS buffer solution (pH = 7.4, Yida Technology (Quanzhou) Co., Ltd., Quanzhou, China), 0.1 mol/L phosphoric acid standard solution (Tanmo Quality Inspection—Standard Substance Center, Shanghai, China), 0.1 mol/L acetic acid standard solution (Dongguan Pinggen Laboratory Equipment Co., Ltd., Dongguan, China), glycerol (analytical grade, relative molecular mass: 92.09; Tianjin Zhiyuan Chemical Reagent Co., Ltd., Tianjin, China), deionized water (provided by Dow Water Treatment Equipment Engineering Co., Ltd., Shanghai, China), KMP-BP-212-37-S (Beijing Kehua Microelectronics Materials Co., Ltd., Beijing, China), SU-8-2150 (Microchem Corporation, Newton, MA, USA), SYLGARD 184 (Dow Corning Corporation, Midland, MI, USA), and a capillary glass tube (Model 3520 rectangular test tube, VitroCom, Mountain Lakes, NJ, USA).

The following experimental instruments were used: a visible spectrophotometer (Model 721G, Shanghai Instrument and Electrical Analysis Instrument Co., Ltd., Shanghai, China), a pH meter (Model PHS-25, Shanghai Instrument and Electrical Scientific Instrument Co., Ltd., Shanghai, China), an electronic scale (Yongkang Airui Trading Co., Ltd., Jinhua, China), a heat-collecting constant-temperature heating magnetic stirrer (Model DF-101S, Tianjin Huaxin Instrument Co., Ltd., Tianjin, China), an imaging system (CCD industrial camera; Shanghai Zhiqi Industrial Co., Ltd., Shanghai, China), and a scanning electron microscope (SEM) (Model 6900, Beijing Zhongke Scientific Instruments Co., Ltd., Beijing, China).

Acquisition and dispersion of MM were carried out as follows: the MMs are composed of an iron–cobalt–nickel alloy. This alloy material was chosen due to its excellent saturation magnetization, which can generate strong magnetic response and driving capability under an external magnetic field, satisfying the motion actuation demand of microswimmers. Microspheres have a particle size range of 150–250 mesh. To obtain MMs with a particle size of less than 75 μm, filtration and screening were conducted using filter cloths with sizes of 100 mesh, 150 mesh, and 200 mesh. The final screened microspheres had particle sizes ranging from 58 μm to 75 μm, with an average particle size of 66 μm. MCMs with a particle size of less than 200 μm were isolated via filtration through 100-mesh and 200-mesh filter cloths. For dispersion treatment, an appropriate amount of microspheres (either MMs or MCMs) was placed in a container with pure glycerol solution and then alternately inverted. By utilizing the gravitational settling effect and liquid surface tension, we achieved uniform dispersion of the microspheres in glycerol.

In the experiment, macroscopic images of the microspheres were captured using a camera. Color images showing the size and morphology of the microspheres were acquired via an optical microscope. Black and white images of the local morphology of microspheres were obtained using an atomic force microscope. When observing microspheres under an optical microscope, the microspheres were placed in a container (or observation cell) with a width of 2 mm and an inner wall height of 0.2 mm.

DOX loading and release experiments of the MCMs were conducted at room temperature in a dark chamber. The samples were briefly gently shaken manually for homogenization every six hours and then stored statically.

The experimental data includes both the directly recorded experimental results and those converted from primary experimental data. The data curves with error bars shown in the figures represent the average values of multiple replicate results. The error bars in this study denote the standard error of the mean. Each group of experiments was repeated no less than three times, and three valid datasets were adopted for statistical analysis. To ensure the objectivity and reproducibility of experimental results, all original data were completely recorded and properly archived.

## 3. Results

### 3.1. Drug Loading and Release of MMs

**Figure 1:**
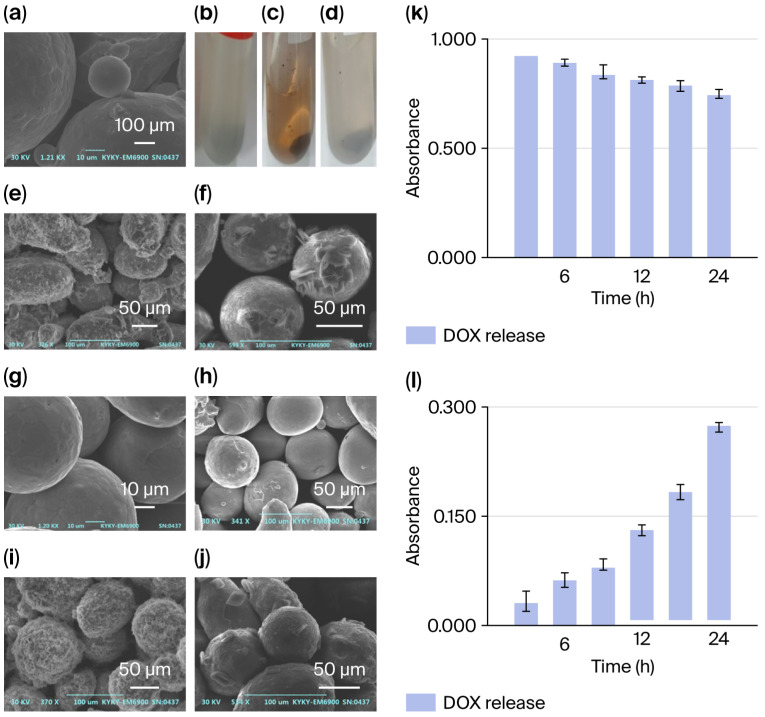
Experimental process of physical adsorption method. (**a**) Microspheres dispersed in ethanol; (**b**,**c**) colors of solutions after microspheres were soaked in PBS buffer and DOX solution for 24 h, respectively. (**d**) Color of pH 5.5 PBS solution with microspheres pre-soaked in DOX aqueous solution. (**e**) Unwashed PBS-soaked microspheres and (**f**) those washed with deionized water. (**g**) Unwashed DOX-soaked microspheres and (**h**) those washed with deionized water. (**i**) Microspheres (first DOX-soaked, then PBS-soaked) unwashed after PBS treatment and (**j**) those washed with deionized water after PBS treatment. (**k**) 24 h absorbance change in DOX solution with immersed microspheres; (**l**) 24 h absorbance change in PBS buffer with DOX-pretreated microspheres.

The microspheres immersed in pH 5.5 PBS for 24 h show substantial morphological changes (Figure 1e,f) compared to the ethanol-dispersed microspheres (Figure 1a). The PBS solution became pale white and turbid post-immersion (Figure 1b), and residual substances remained on the microspheres even after thorough washing with deionized water.

#### 3.1.1. Morphological Comparison of Microspheres Under Different Treatments

During drug loading (microspheres immersed in DOX solution), solution absorbance was recorded at 3 h intervals, and the absorbance–soaking time correlation is shown in Figure 1k. Similarly, during drug release (microspheres in pH 5.5 PBS buffer), supernatant absorbance was measured every 3 h, and the corresponding relationship is presented in Figure 1l.

No significant morphological changes were observed in microspheres soaked in DOX solution compared to ethanol-dispersed ones (Figure 1a). The DOX solution color (Figure 1c) and microsphere morphology (Figure 1g,h) are shown.

Microspheres pretreated with DOX (soaked, dried) then immersed in pH 5.5 PBS exhibited pronounced surface morphological changes (Figure 1i,j) compared to ethanol-dispersed ones (Figure 1a). The solution color is shown in Figure 1d, with residual deposits remaining after washing with deionized water.

Microspheres soaked in pH 5.5 PBS alone (Figure 1b,e,f) and those first soaked in DOX then PBS (Figure 1d,i,j) show striking similarities: both solutions were white and turbid, and the microspheres had rough surfaces before washing with deionized water, with minimal residuals afterward.

#### 3.1.2. Experimental Data Analysis of Microspheres Under Different Treatments

The experimental data showed that the average absorbance change in solutions containing soaked microspheres was lower than that of the pH 5.5 PBS solutions loaded with DOX-treated microspheres. This discrepancy is primarily caused by the reaction between the microspheres and phosphate species in an acidic PBS environment. Two pieces of evidence support this conclusion: first, the alloy microspheres react with dilute phosphate solution to generate insoluble iron phosphate and cobalt phosphate precipitates; second, the color of soaking solution remains consistent regardless of whether the microspheres are preloaded with DOX or not.

It should be noted that iron–cobalt–nickel alloy microspheres exhibited poor chemical stability under acidic conditions, as the alloy components react with phosphate ions and produce insoluble precipitates. Evident surface corrosion and structural shedding of microspheres were observed through microscopic characterization. These degradation by-products interfere with visible spectrophotometry detection. Therefore, the measured absorbance variation cannot fully represent the actual released DOX content, which imposes limitations on the quantitative analysis of drug release behavior. This limitation has been clearly illustrated in the manuscript.

In summary, while MMs can directly physically adsorb some DOX, reaction by-products in pH 5.5 PBS (mimicking the acidic microenvironment of cancer cells) inevitably interfere with DOX absorbance detection. This interference makes it unfeasible to accurately quantify the actual DOX release kinetics, so unmodified MMs are ill-suited for direct use as drug carriers.

### 3.2. Preparation and Drug Loading Analysis of MCM

This subsection explores the chitosan coating method for comparative study, aiming to evaluate its applicability and screen out reliable preparation strategies for drug delivery.

#### 3.2.1. CS Method

**Figure 2:**
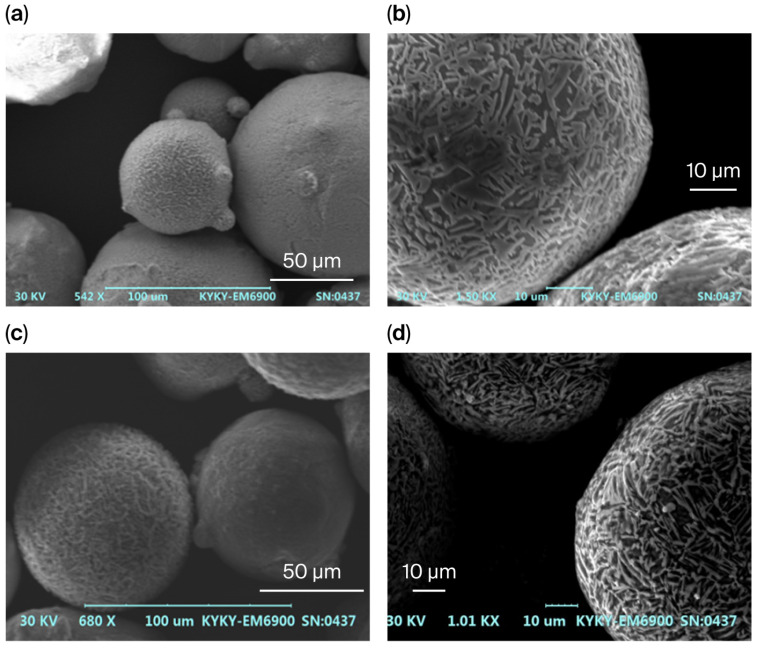
SEM morphologies of the test samples after soaking in different solutions. (**a**) MMs soaked in chitosan aqueous solution for 24 h; (**b**) MMs immersed in CS for 72 h; (**c**) MMs immersed in acetic acid solution for 24 h; (**d**) MMs immersed in acetic acid solution for 72 h.

To prepare MCMs using CS, 4 g of chitosan powder was added to 200 mL deionized water, and then acetic acid was added dropwise while stirring magnetically. The pH was adjusted to below 6.4, until the chitosan dissolved completely. Microspheres were added to CS and stirred for even dispersion, and then NaOH was added and mixed quickly. The precipitate was collected, washed thoroughly with deionized water, and dried (the product is shown in Figure 2a). Extending the microspheres’ soaking time in CS to 72 h yielded the product shown in Figure 2b.

Compared with untreated microspheres (pristine, smooth; Figure 1a), acetic acid-treated ones (Figure 2c) and those in acetic acid-supplemented chitosan aqueous solution (Figure 2a) showed similar substantial surface transformations, indicating that surface alterations are mainly caused by acetic acid corrosion. Extending the soaking time in both solutions led to further surface morphology evolution (Figure 2b,d) with more pronounced, deeper indentations, confirming that the irregular microsphere surfaces result from acetic acid corrosion.

#### 3.2.2. CC Method

**Figure 3:**
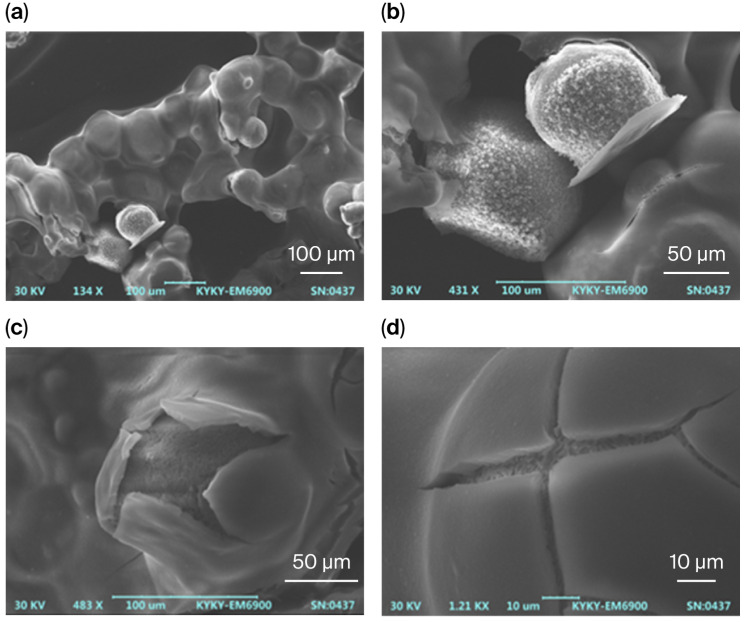
SEM morphology of microspheres soaked in CC solution. (**a**) CC-encapsulated microspheres; (**b**–**d**) show microspheres with chitosan layers of different cracking degrees.

Preparation of MCMs using CC was carried out as follows: 4 g of chitosan was weighed and placed into a beaker using a microelectronic balance, mixed with 250 mL of acetic acid solution, and stirred continuously for 12 h with a magnetic stirrer until fully dissolved to obtain CC. An appropriate amount of microspheres was dispersed in CC via thorough shaking, and the resultant microsphere-containing CC solution was air-dried to yield chitosan-encapsulated MMs (Figure 3).

Microspheres directly dried from CC were tightly wrapped in chitosan; individual MMs were either fully encapsulated or randomly cracked to varying degrees. Higher CC concentrations enhanced the post-drying toughness of the colloid. Despite the tight MM–chitosan adhesion, grinding failed to disrupt and disperse the chitosan matrix—likely owing to the strong internal bonding and retained elasticity of dehydrated CC, which resisted mechanical squeezing. Thus, direct drying of CC impeded the preparation of well-dispersed chitosan-coated microspheres. The aggregation defect indicates that this coating strategy cannot meet the experimental requirements, which lays a foundation for subsequent scheme improvement.

**Figure 4:**
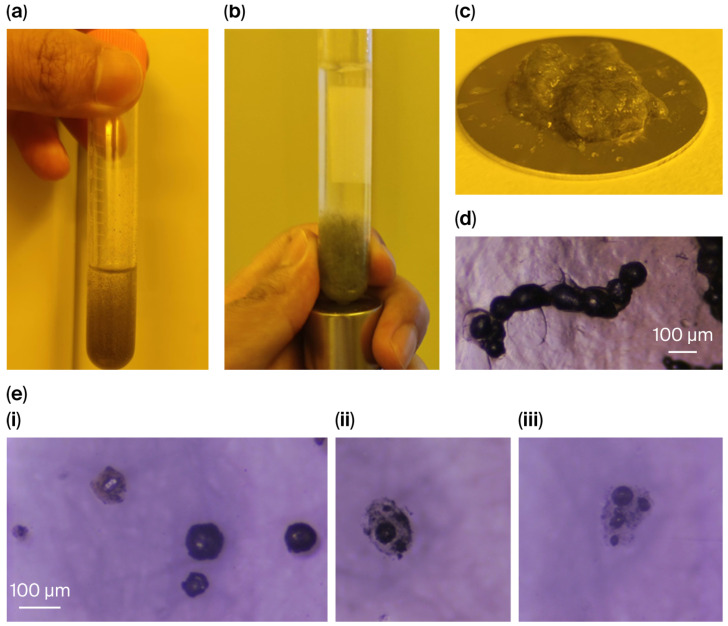
Preparation of MCMs by adding NaOH to CC. (**a**) Dispersion of MMs in CC; (**b**) separation after precipitation of magnetic microsphere-based CG; (**c**) magnetic microsphere-based CG washed with deionized water; (**d**) morphology of dried chitosan–magnetic microsphere complex; (**e**) MCMs viewed under optical microscope: (i) bare magnetic microspheres without chitosan coating, (ii) single magnetic microsphere encapsulated MCM, (iii) MCM containing multiple embedded magnetic microspheres.

Microspheres were thoroughly dispersed in chitosan solution (CS, Figure 4a), and then NaOH solution was added dropwise with vigorous shaking after each addition until complete CC coagulation was achieved. The resulting chitosan-coated microsphere gel was repeatedly washed with deionized water while the CG was immobilized with permanent magnets (Figure 4b). The washed CG is presented in Figure 4c. The coated-microsphere CG was dried at 45 °C; once the surface moisture evaporated, it was cut and mechanically dispersed, and drying was continued in a constant-temperature oven. Finally, the fully dried gel was ground with a mortar and filtered through a 100-mesh cloth, and the screened product was dispersed in glycerol (Figure 4e).

As shown in Figure 4c, adding NaOH to CC yields a gel with dispersed MMs; however, the MMs are relatively concentrated in the gel shown in Figure 4d, and few microspheres remained encapsulated by chitosan after grinding and dispersion (Figure 4e). Notably, the chitosan layer is more likely to encapsulate smaller microspheres (Figure 4e(iii)). MMs were smaller than 75 μm in size, while the size of MCMs remained below 200 μm. A single MM occupies no more than half the volume of an MCM particle, and plenty of irregular fragments were found to be randomly distributed inside MCMs. From Figure 4e(i), many exposed MMs can be observed in the dispersion system with irregular surface morphology. Combined with the distribution characteristics in Figure 4e, the fine fragments inside MCMs were confirmed to stem from MMs damaged by extrusion during the grinding process.

Due to the high viscosity of CC, microspheres do not settle shortly after dispersion. When NaOH solution is added and shaken, it fails to diffuse uniformly in the colloid, leading to irregularly shaped, large-sized chitosan precipitates. Additionally, during CG precipitation, microspheres settle naturally and tend to aggregate, thus being wrapped together by CC. Moreover, grinding compresses the dried chitosan–magnetic microsphere complex, causing simultaneous chitosan fragmentation and microsphere dispersion. This leaves little or no chitosan on individual microsphere surfaces (Figure 4e). Therefore, when preparing MCMs by adding NaOH to microsphere-dispersed CC in a dropwise manner, the yield of microspheres fully coated with a chitosan layer is relatively low. As low yield and severe aggregation restrict the practical application of this method, further process improvement is essential.

#### 3.2.3. Optimized Process

**Figure 5:**
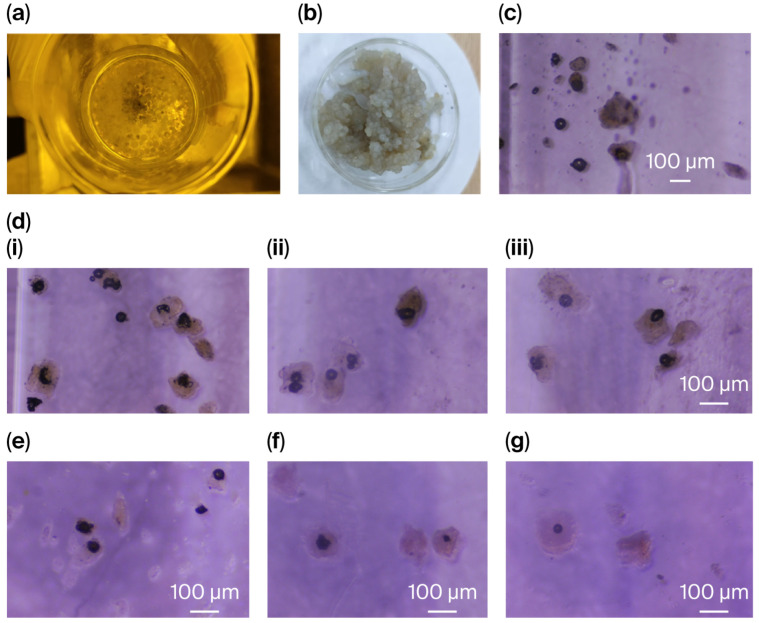
Morphological changes in MCMs (prepared by adding CC to NaOH solution) before and after drug loading. (**a**) Magnetic microsphere-based CG precipitated in NaOH solution; (**b**) magnetic microsphere-based CG after washing with deionized water; (**c**) MCMs prepared by one-time grinding and dispersion; (**d**) MCMs prepared using improved process: (i) five discrete MCM particles, each consisting of one magnetic microsphere encapsulated in chitosan; (ii) four such MCM particles; (iii) three such MCM particles; (**e**) no treatment; (**f**) after DOX soaking; (**g**) after PBS immersion treatment.

Without changing the CC preparation method, the MM-containing CC solution was subjected to reciprocating oscillation until the microspheres were fully dispersed. Then, the solution was rapidly poured into the NaOH solution while stirring continuously with a glass rod to allow CC to precipitate at the bottom of the beaker (a spherical CC precipitate is shown in Figure 5a). Next, the microsphere-coated CG was thoroughly washed with deionized water (Figure 5b) and, after surface drying, it was refined and dispersed via mechanical cutting, constant-temperature drying, and grinding. The final ground dispersion is presented in Figure 5c.

As seen in Figure 5c, preparing MCMs by adding CC to NaOH solution increases the number of chitosan-encapsulated microspheres. However, abundant dispersed chitosan fragments were observed around the chitosan–magnetic microsphere complex. This indicates that mechanical grinding and dispersion of the dried complex rely on grinding-induced squeezing, but excessive grinding force and secondary grinding/squeezing of already-dispersed MCMs can cause chitosan layer fragmentation.

With the chitosan precipitation mode unchanged, its dispersion method was optimized as follows: water-washed magnetic microsphere-based CG was heat-deionized at 60 °C; once surface moisture evaporated, it was pre-dispersed via mechanical cutting and reheated. The cycle of evaporation, cutting/dispersion, and reheating was repeated until the gel was sufficiently fine. Subsequently, the dispersed magnetic chitosan particles were dried at constant temperature. Post-drying, the particles were pre-filtered with a 100-mesh cloth and the filtrate was collected; the unfiltered particles were ground in stages (controlling grinding force) and filtered after each round. The MCMs that passed through the cloth were collected, and the residue was re-ground until full filtration was achieved. The final dispersion of the chitosan–magnetic microsphere complex is shown in Figure 5d.

Compared with Figure 5c, it can be seen that optimized dispersion and grinding processes improve the yield and coating quality of chitosan-modified microspheres, and they also show a relatively uniform size. Meanwhile, there is an obvious increase in the quantity of MMs fully wrapped by chitosan. The characteristic size of the prepared MCMs is about 150 μm. Internal MMs are discretely distributed with similar diameters, and the size of a single microsphere is approximately half that of MCM particles. Fine impurities on the interior and surface of MCMs are greatly reduced. The uniform and compact chitosan coating indicates that the MMs maintain a complete structure without experiencing damage in the grinding process. Although this optimized method obtains better coating performance, residual free chitosan fragments can still be seen. A uniform shell structure on the microsphere surface was not fully verified through characterization tests under the current conditions.

To verify the drug loading effect of MCMs, the untreated MCMs were first dispersed (morphology shown in Figure 5e). Then, the MCMs were immersed in a DOX aqueous solution of a specific concentration for 24 h (the morphology of the resulting microspheres is shown in Figure 5f). The DOX-soaked MCMs were washed with deionized water and then immersed in pH 5.5 PBS buffer solution for 24 h (the morphology of the obtained microspheres is shown in Figure 5g).

A comparison of Figure 5e and Figure 5f reveals that the chitosan layer on the microspheres darkened markedly after DOX solution immersion, indicating that the MCMs had effectively adsorbed DOX. Comparing Figure 5f and Figure 5g reveals that MCMs pre-soaked in DOX solution faded after immersion in pH 5.5 PBS, suggesting partial release of DOX adsorbed on the chitosan layer. Thus, the prepared MCMs can load DOX and release it in a weakly acidic environment.

**Figure 6:**
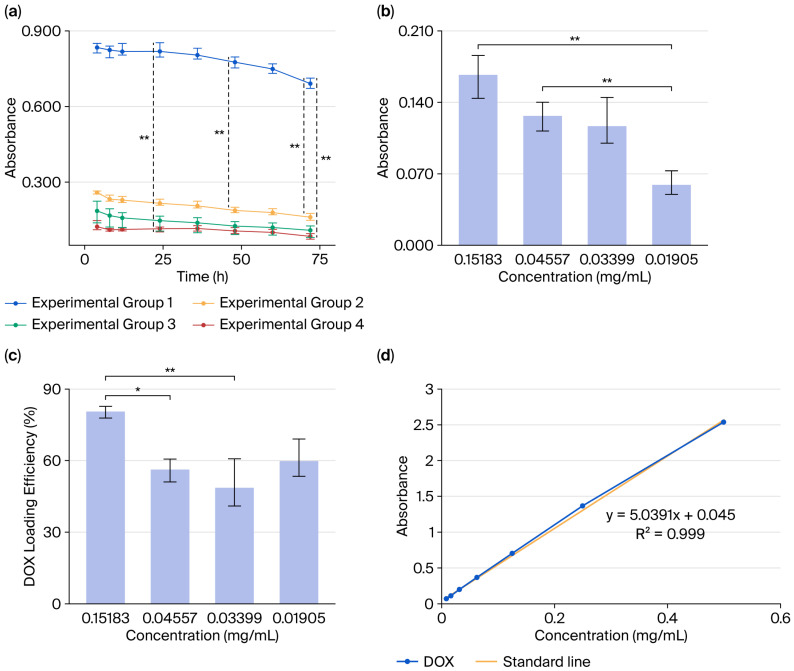
DOX loading onto MCMs. (**a**) Relationship between DOX solution soaking time and absorbance; (**b**) relationship between DOX solution concentration and cumulative absorbance change; (**c**) relationship between DOX solution concentration and drug loading efficiency. (**d**) Standard curve with R^2^ value. Data are shown as mean ± SD, n = 3. Statistical differences were assessed using Student’s *t*-test. * *p* < 0.05; ** *p* < 0.01.

To further quantify the DOX loading characteristics of MCMs, drug release tests were performed on DOX-loaded MCMs using four PBS solutions with different pH values. DOX solutions were prepared at concentrations of 0.152, 0.046, 0.034, and 0.019 mg/mL, designated Concentrations 1–4 in descending order. Ten milliliters of each DOX solution was used as the control group. Sixteen equal aliquots of magnetic chitosan microsphere samples were assigned to four parallel groups per concentration (Experimental Groups 1–4, *n* = 4). Solution absorbance was recorded over 72 h: at 4 h intervals for the first 12 h, then every 12 h thereafter. The absorbance–soak time relationship is shown in Figure 6a, the 72 h cumulative absorbance change vs. DOX concentration is shown in Figure 6b, the 72 h drug loading efficiency vs. DOX concentration is shown in Figure 6c, and the standard curve correlating doxorubicin concentration and absorbance is shown in Figure 6d. The DOX loading efficiency of MCMs is

(1) $$\eta_{d\_l}=\frac{W_{l}}{W_{t}}\times 100\%$$

where $W_{t}$ is the total weight of DOX in the solution before DOX is loaded onto the MCMs, and $W_{l}$ is the weight of DOX loaded onto the MCMs.

Figure 6a shows that the absorbance of all four experimental groups (with varying DOX concentrations) decreased over the 72 h soaking period. Figure 6b indicates that the 72 h cumulative absorbance change in DOX solutions with MCMs rises with an increasing DOX concentration, with values of 0.167, 0.127, 0.117, and 0.059 corresponding to concentrations of 0.152, 0.046, 0.034, and 0.019 mg/mL, respectively. Meanwhile, Figure 6c shows that the MCM DOX loading efficiency increases with DOX concentration, reaching 80.57%, 56.14%, 48.56%, and 59.69% at the respective concentrations mentioned above.

Furthermore, Figure 6c shows that MCMs have higher DOX loading efficiency at 0.019 mg/mL than at 0.046 and 0.034 mg/mL. This phenomenon is thought to stem from structural differences among samples. It is inferred that samples obtained under low drug concentration may achieve relatively better chitosan encapsulation, while extra unbound chitosan exists in the other two groups, which may improve the drug adsorption performance. Nevertheless, no TEM observation or dye exclusion test was conducted to verify coating uniformity, and a relevant statistical analysis is also absent. The above explanation is only a reasonable deduction based on experimental trends.

For the drug release test, PBS buffers with pH of 7.4, 6.5, 5.5, and 4 were prepared, and cumulative DOX release from drug-loaded MCMs was monitored over 96 h, with absorbance was measured every 4 h for the first 12 h and then every 12 h. Four MCM samples (equal to those in the DOX loading experiment) were mixed with 10 mL of PBS (sorted by increasing pH) to form Control Groups 1–4. For the experimental groups (MCM pre-soaked in Concentration 1/2 DOX solutions for 72 h), the DOX solution was discarded, the microspheres were rinsed with deionized water, and then 10 mL of the four pH-gradient PBS solutions were added to the test tubes of the two concentration groups (with four replicates per concentration).

**Figure 7:**
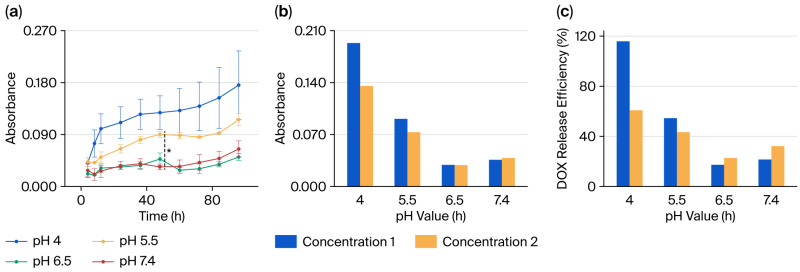
DOX release onto MCMs. (**a**) Relationship between PBS solution soaking time and absorbance; the black dotted line at 48 h denotes a statistically significant difference between the pH 5.5 and pH 7.4 groups; (**b**) relationship between PBS solution pH value and absorbance; (**c**) relationship between PBS solution pH value and DOX release efficiency. Data are shown as mean ± SD, n = 3. Statistical differences were assessed using Student’s *t*-test. * *p* < 0.05.

Absorbance at different pH values was recorded over 96 h (every 4 h for the first 12 h and then every 12 h), with the mean of duplicate measurements calculated. The cumulative absorbance change after 96 h of PBS immersion was defined as the DOX release amount of MCMs (pre-soaked in DOX solution for 72 h). MCM DOX release efficiency was determined from two parameters: the 96 h PBS release amount and the 72 h DOX soaking-induced cumulative absorbance change. The results are shown in Figure 7. The DOX release efficiency of MCM is

(2) $$\eta_{d\_r}=\frac{W_{r}}{W_{l}}\times 100\%$$

where $W_{r}$ is the weight of DOX released in the PBS solution.

Figure 7a shows that, in the 96 h DOX release experiment, the absorbance of PBS solutions at different pH values all increase with prolonged soaking time.

Figure 7b shows that, after 96 h of immersion of drug-loaded MCMs in PBS, the solution’s absorbance decreases with increasing pH. At the same pH, Concentration 1 exhibits higher absorbance than Concentration 2, with this difference being more pronounced at lower pH.

The reasons for this are as follows: first, an acidic environment with a lower pH speeds up the corrosion and degradation of microcarriers, facilitating the outflow of loaded DOX; second, considering the results in Figure 7c, the sample group with Concentration 1 possesses relatively high drug loading capacity, thus presenting a large cumulative release amount. The calculated release efficiency reaches 115.74% at pH 4. This abnormal phenomenon is attributed to an imperfect chitosan encapsulation structure. A partially exposed magnetic matrix reacts with phosphate solution under acidic conditions, generating colored by-products. Such substances will interfere with ultraviolet absorbance signals and cause the measured absorbance value to be higher than the actual concentration of released DOX. It is hard to completely distinguish the signal contributions of the target drug and corrosion products with the available means of detection.

Figure 7c shows that the DOX release efficiency of MCMs increases with decreasing pH, with the Concentration 1 group exhibiting higher efficiency than Concentration 2. In contrast, Figure 7b reveals that at pH 6.5 and 7.4, the cumulative DOX release amount and the efficiency of drug-loaded MCMs are comparable, while Concentration 1 shows lower values than Concentration 2 at pH 6.5.

The underlying reasons are as follows: in weakly acidic PBS environments (pH 7.4 and 6.5), acid has no significant effect on MCMs and DOX release mainly relies on free diffusion in PBS, resulting in relatively low and similar cumulative release amounts and efficiencies. The lower release in Concentration 1 may stem from fewer MMs being encapsulated by chitosan in equal-weight samples, whereas Concentration 2 contains more chitosan unencapsulated by MMs.

**Figure 8:**
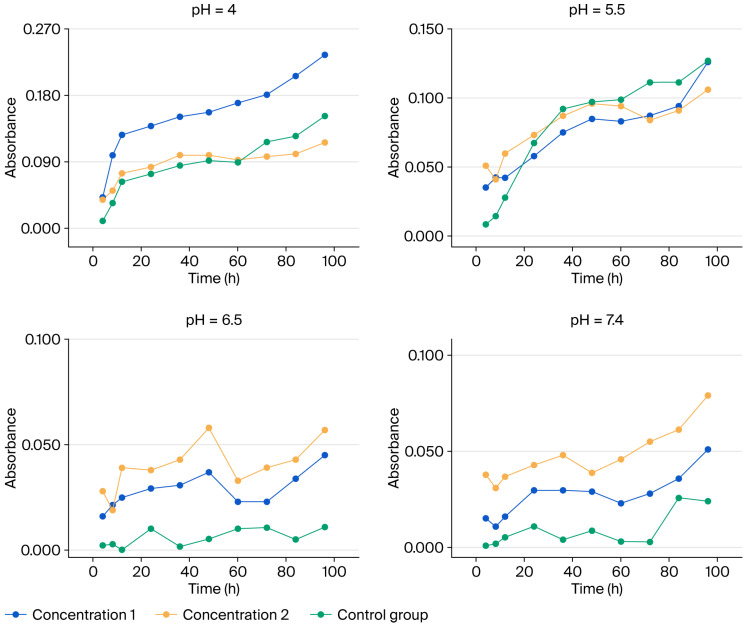
The correlation between soaking time and absorbance values across varying pH conditions.

DOX-unloaded MCMs served as the control group, while MCMs loaded with DOX at Concentrations 1 and 2 were the experimental groups. The correlation between soaking time and absorbance after 96 h of immersion in pH-gradient PBS solutions is presented in Figure 8.

Concentration 1 consistently showed higher absorbance than Concentration 2, owing to its greater encapsulated DOX content and subsequent higher cumulative release. Notably, in pH 5.5 PBS, the control group’s absorbance surpassed that of both experimental groups after 24 h of incubation. This is likely due to incomplete or absent chitosan encapsulation of MMs in all samples: prolonged acidic exposure triggers reactions between the exposed metallic MM surface and phosphoric acid, and the resulting colored products mask the absorbance contribution from DOX release.

Furthermore, the control group exhibited distinct pH-dependent time-course absorbance profiles, with marked variations at pH 4 and 5.5 but minimal changes at pH 6.5 and 7.4—indicating that its absorbance fluctuations were primarily derived from chemical interactions between the metallic microsphere surface and weak acids. In contrast, the absorbance change mechanisms of Concentrations 1 and 2 were pH-dependent. At pH 7.4 and 6.5, their absorbance remained consistently higher than that of the control group, implying that DOX-free diffusion dominated the changes. At pH 5.5 and 4, both experimental groups and the control group showed substantial temporal absorbance changes, reflecting the dominant role of sample–solution interactions—chitosan layer dissolution and subsequent DOX release for the experimental groups, and chemical reactions between the magnetic microsphere metal layer and dilute phosphoric acid for the control group.

The blank control group without loaded DOX also exhibited obvious absorbance fluctuation in an acidic environment. This result further confirms that corrosion-generated colored by-products can greatly interfere with visible light detection signals. The measured absorbance signal is the superposition of released doxorubicin and interfering substances, rather than being purely derived from drug release.

The coating efficiency of the chitosan shell exerts obvious regulatory effects on drug loading and release properties of magnetic microspheres. A higher coating density contributes to a denser outer shell structure and greater diffusion resistance, which inhibits initial burst release and prolongs the drug release period. Low coating efficiency weakens the barrier protection, leading to rapid drug dissolution and poor release stability. Additionally, the coating layer mitigates the impact of an environment with medium acidity, and differences in coating degree ultimately alter the overall release rate and cumulative release amount.

## 4. Discussion

Two distinct drug carriers were used in the drug delivery investigation. The microsphere morphology was systematically analyzed via four preparation methods, and the drug loading performance of the carriers was quantified, with the results summarized in Table 1.

The following conclusions are drawn regarding the microsphere drug loading process:

- Iron–cobalt–nickel microspheres readily undergo chemical reactions with phosphoric acid in PBS, and the colored reaction products severely interfere with experimental analysis, making these microspheres unsuitable for direct use as drug carriers.
- Diluting CS with water significantly reduces its viscosity; meanwhile, microspheres sediment faster due to gravity. Consequently, chitosan precipitates easily upon dropwise addition of NaOH solution, hindering the synthesis of MCMs.
- CC inherently possesses high viscosity and, following natural drying, retains a considerable degree of elasticity. These physical properties pose significant challenges to mechanical dispersion, thereby complicating the preparation of MCMs.
- Introducing MM-pre-dispersed CC into the NaOH solution causes the formation of spherical CG encapsulating microspheres. However, high CC viscosity and liquid surface tension result in relatively large CG spheres when using the dropping method, necessitating subsequent secondary mechanical cutting and dispersion to obtain discrete MCMs.
- MCMs can effectively load and release DOX molecules, with release efficiency strongly correlated with PBS pH and soaking duration—specifically, lower pH and longer soaking times yield higher release efficiency.

Corrosion and degradation are common phenomena for metal-based micromotors in biological media, which greatly affect structural stability and experimental reliability [41,42,43]. As reported in existing studies, iron–cobalt–nickel alloys tend to undergo electrochemical corrosion and coordination reactions in acidic environments containing phosphate ions. This leads to rough surface morphology, structural shedding, and formation of insoluble phosphate corrosion products. In this study, corrosion damages the integrity of the coating layer and weakens drug loading stability. Corrosion impurities also interfere with visible spectrophotometric measurement, causing deviation in drug release absorbance data. Moreover, changes in surface morphology and material composition induced by corrosion will further impair the movement performance and working stability of micromotors. Accordingly, environmental corrosion is an inevitable limiting factor for biomedical drug delivery micromotor systems.

Chitosan serves as a classic material for constructing drug-carrying micromotors and surface functional layers, and many related research achievements have been reported [44,45]. Distinct from previously developed chitosan-based micromotor platforms, this work employs magnetic Fe-Co-Ni alloy microspheres as the driving substrate that can respond to a gradient magnetic field. Through successive optimization of coating parameters, the prepared composite microswimmer effectively balances the magnetic actuation capacity and shell protection effect. Compared with existing designs, this study optimizes the preparation route to alleviate corrosion-induced structural damage and realize stable drug loading on magnetic microcarriers. The integrated design provides a differentiated technical idea for the development of biomedical magnetic drug delivery micromotors.

## 5. Conclusions

This study investigated the drug loading capacity and release properties of MMs and chitosan-modified MMs. Two methods (CS and CC) were used to prepare MCMs. CS failed to form chitosan shells on MMs, whereas CC enabled the successful fabrication of chitosan-coated MMs. Two CC-based MCM preparation approaches were tested—namely, adding NaOH to MM-dispersed CC and adding MM-dispersed CC to NaOH—with the latter yielding more MCMs with uniform chitosan coatings. Optimizing the cutting, heating, grinding, and dispersion processes during MM and CG encapsulation further improved the quality and yield of MCMs. Drug loading and release experiments showed that unmodified MMs were unsuitable for direct drug loading, while chitosan-modified MMs effectively loaded and released DOX. In summary, this study proposes a novel surface modification strategy for micro-robots, facilitating cost-effective drug delivery using micro-nano robots. However, challenges persist; in particular, the incomplete uniform dispersion of MMs in CC and high CC viscosity hinder the preparation of monodispersed MCMs, with single MMs encapsulated in individual chitosan spheres. Additionally, the thickness and uniformity of the chitosan shells in the resultant MCMs require further optimization.

## Figures and Tables

**Table 1:** Comparative analysis of different schemes and processes.

Material	Preparation Method	Feature	Drug Loading Efficiency
MM	Obtained directly	The surface is rough after soaking in PBS ^1^	127.00%
MCM	Immersion in chitosan aqueous solution	The surface of the MM is smooth	-
MCM	NaOH is added to the CC solution	Chitosan layers can be obtained, but they are prone to cracking and difficult to disperse after drying	-
MCM	CC is added to the NaOH solution	Chitosan-coated MMs can be obtained, but in small quantities	-
MCM	Immersed in solution and ground multiple times	Chitosan-coated microspheres can be obtained, and the yield is relatively increased	115.74%

^1^ The pH value of the PBS solution is 5.5.

## Data Availability

The original contributions presented in this study are included in the article. Further inquiries can be directed to the corresponding author.

## Abbreviations

The following abbreviations are used in this manuscript:

DOX	Doxorubicin
MMs	Magnetic microsphere
CS	Chitosan solution
CC	Chitosan colloid
CG	Chitosan gel
MCMs	Magnetic chitosan microsphere
